# Aspirin and Cancer Survival: An Analysis of Molecular Mechanisms

**DOI:** 10.3390/cancers16010223

**Published:** 2024-01-03

**Authors:** Manoj Pandey, Monika Rajput, Pooja Singh, Mridula Shukla, Bin Zhu, Jill Koshiol

**Affiliations:** 1 Department of Surgical Oncology, Institute of Medical Sciences, Banaras Hindu University, Varanasi 221005, India; monika05rajput@gmail.com (M.R.);; 2 RRL, Dr. Lalpath Labs Ltd., Shivpur, Varanasi 221003, India; 3 Division of Cancer Epidemiology and Genetics (DCEG), National Cancer Institute (NCI), National Institutes of Health (NIH), 9609 Medical Center Drive, RM 6-E212, Rockville, MD 20850, USAkoshiolj@mail.nih.gov (J.K.)

**Keywords:** aspirin, COX2, mechanism, cancer survival, pathway, genes, proteins

## Abstract

**Simple Summary:**

The use of aspirin has shown a definite role in the prevention of cancer; however, its effect on survival is still debated. The evidence from randomized trials failed to show any benefit, while cohort studies have demonstrated its usefulness. This is attributed to the use of different doses of aspirin in differing studies. This article explores the plausible mechanisms through which aspirin may exert its effect on improving survival. It is possible that the use of aspirin as adjunct to standard care may lead to better survival in cancer, though the actual effect would have to be demonstrated in clinical trials.

**Abstract:**

The benefit of aspirin on cancer survival is debated. Data from randomized clinical trials and cohort studies are discordant, although a meta-analysis shows a clear survival advantage when aspirin is added to the standard of care. However, the mechanism by which aspirin improves cancer survival is not clear. A PubMed search was carried out to identify articles reporting genes and pathways that are associated with aspirin and cancer survival. Gene ontology and pathway enrichment analysis was carried out using web-based tools. Gene–gene and protein–protein interactions were evaluated. Crosstalk between pathways was identified and plotted. Forty-one genes were identified and classified into primary genes (*PTGS2* and *PTGES2*), genes regulating cellular proliferation, interleukin and cytokine genes, and DNA repair genes. The network analysis showed a rich gene–gene and protein–protein interaction between these genes and proteins. Pathway enrichment showed the interleukin and cellular transduction pathways as the main pathways involved in aspirin-related survival, in addition to DNA repair, autophagy, extracellular matrix, and apoptosis pathways. Crosstalk of *PTGS2* with *EGFR*, *JAK/AKT*, *TP53*, interleukin/*TNFα*/*NFκB*, *GSK3B/BRCA/PARP*, *CXCR/MUC1*, and *WNT/CTNNB* pathways was identified. The results of the present study demonstrate that aspirin improves cancer survival by the interplay of 41 genes through a complex mechanism. *PTGS2* is the primary target of aspirin and impacts cancer survival through six primary pathways: the interleukin pathway, extracellular matrix pathway, signal transduction pathway, apoptosis pathway, autophagy pathway, and DNA repair pathway.

## 1. Introduction

Despite accumulating evidence of the benefit of aspirin against cancer, its effect on improving cancer survival is still debated, since the mechanism by which it impacts cancer survival is not completely understood and the published data are discordant. There have been four randomized controlled trials (RCTs) [1,2,3,4] showing mixed results from no effect to improved survival. Lipton et al. reported the first randomized trial in 1982 in 66 patients with Duke’s B or C, colon, and rectal cancers, randomized to 600 mg of aspirin or placebo for two years, and demonstrated no difference in disease-free or overall survival rates [3]. A second RCT in 1991 randomized patients with renal cancer to interferon and interferon plus 600 mg of aspirin and reported better results with interferon alone [1]. The third randomized study, conducted in 1993, randomized patients with lung cancer to chemotherapy or chemotherapy with aspirin. In this trial, a daily dose of 1000 mg of aspirin was used, because this dose is supposed to influence platelet function. However, this study also failed to show any benefit of adding aspirin to the standard of care [2]. All these three trials used a relatively high dose of aspirin, ranging from 600 mg to 1000 mg. However, in 2009, Liu et al., for the first time, reported improved survival in esophageal cancer patients randomized to a very low dose of aspirin (50 mg) [4]. Since then, several retrospective and observational studies have reported a survival advantage of adding aspirin to the treatment for various cancers. A meta-analysis of 118 studies, 63 of them specifically reporting on cancer mortality and the rest on all-cause mortality, found a 21% reduction in cancer deaths and about 20% reduction in all-cause mortality (pooled hazard ratio (HR): 0.79; 95% confidence intervals: 0.73, 0.84) [5].

All the studies reported increased instances of bleeding in patients taking aspirin. However, this bleeding was not fatal in any of the cases. It has also been observed that the benefit appears in all cancers and is not limited to any cancer in particular. For example, aspirin use has been associated with improved survival for colorectal cancer (HR = 0.38; 95% CI = 0.17–0.87; *p* = 0.02) [6], biliary tract cancer [7,8], breast cancer (adjusted HR = 0.69, 95% CI 0.47–0.98) [9], prostate cancer (adjusted hazard ratio, 0.43; 95% CI, 0.21 to 0.87; *p* = 0.02) [10], and Endometrial cancer (hazard ratio 0.46, 95% CI 0.25–0.86, *p* = 0.014) [11].

Various mechanisms have been proposed to explain the protective role of aspirin against cancer. The inhibition of COX2, regulating apoptosis, and reduction in angiogenesis through the prostaglandin pathway are predominant; however, aspirin is also said to improve survival by regulating platelet function and reducing metastasis [12,13]. Other proposed mechanisms include the inhibition of the peroxisome proliferator-activated receptor (PARP) and hence interference with the homologous recombinant (HR) DNA repair pathway, nuclear factor-kB (NFκB), and PI3KC pathway regulation. However, no bioinformatic studies have evaluated the interaction of genes associated with cancer survival and their protein products to investigate the possible mechanism by which aspirin can affect cancer survival. Therefore, we conducted this systematic review and bioinformatic analysis to explore the possible mechanism(s) by which the addition of aspirin may produce a survival benefit in cancer patients.

## 2. Material and Methods

A PubMed search was carried out filtering for English-language papers and using the string ((“aspirin”[MeSH Terms] OR “aspirin”[All Fields] OR “aspirins”[All Fields] OR “aspirin s”[All Fields] AND (“cancer s”[All Fields] OR “neoplasms”[MeSH Terms] OR “neoplasms”[All Fields] OR “cancer”[All Fields] OR “cancers”[All Fields]) AND (“mortality”[MeSH Subheading] OR “mortality”[All Fields] OR “survival”[All Fields] OR “survival”[MeSH Terms] OR “survivability”[All Fields] OR “survivable”[All Fields] OR “survivals”[All Fields] OR “survive”[All Fields] OR “survived”[All Fields] OR “survives”[All Fields] OR “surviving”[All Fields]) AND (“genes”[MeSH Terms] OR “genes”[All Fields] OR “gene”[All Fields] OR (“gene s”[All Fields] OR “genes”[MeSH Terms] OR “genes”[All Fields]) OR (“genome”[MeSH Terms] OR “genome”[All Fields] OR “genomes”[All Fields] OR “genome s”[All Fields] OR “genomically”[All Fields] OR “genomics”[MeSH Terms] OR “genomics”[All Fields] OR “genomic”[All Fields])) to identify articles reporting on genes and pathways that are associated with aspirin and cancer survival. Studies reporting on the molecular mechanism either in clinical or experimental studies on cell lines were evaluated. The search was restricted to English. Non-English studies and studies reporting on NSAID’s other than aspirin or aspirin in combination with other drugs were excluded. The genes were identified, and bioinformatic analysis was performed.

WEB-based Gene Set Analysis Toolkit (Webgestalt) (http://www.webgestalt.org/) was used to perform gene ontology (GO) and pathway enrichment analysis, and GeneMANIA (https://genemania.org) was used for gene–gene interactions. A protein–protein interaction network (PPI) was constructed using NetworkAnalyst (http://www.networkanalyst.ca), and interacting genes were searched using Search Tool for the Retrieval of Interacting Genes (STRING) (http://string-db.org/). Pathways were created using Reactome (https://reactome.org/PathwayBrowser/), and the rest of the data were manually curated using public databases. Crosstalk between enriched pathways was built using the ShinyGO v0.741 tool (http://bioinformatics.sdstate.edu/go74/). Based on a false discovery rate (FDR) *p* value of <0.05, the top 30 pathways were enriched. Using the p values from these 30 pathways, a hierarchical clustering tree was constructed. Crosstalk between the genes was prepared manually from the network. All the above websites were accessed on 20 August 2022.

## 3. Results 

The PubMed search resulted in 189 articles, of which 13 clinical and 32 cell line articles were included to identify genes that are associated with aspirin and cancer survival. Forty-one genes were identified and were grouped into four categories based on their function (Table 1). The first group includes primary genes controlled directly by aspirin, i.e., *PTGS2* and *PTGES2*. The second group consists of genes involved in cell signaling and cell proliferation like *cMyc*, *EGFR*, *BCL2*, *WNT*, *KRAS*, *WNT6*, *BRAF*, *MUC1*, *PIK3CA*, *PARP1*, *PARP2*, *STAT3*, *MAPK*, *JAK/STAT*, and BAX [6,14,15,16,17,18,19,20,21,22,23,24,25,26,27,28,29,30,31,32]; the third group includes genes for cytokines or their receptors (e.g., *IFNγ*, *IL1β*, *IL2*, *IL4*, *IL5*, *IL6*, *IL7*, *IL8*, *IL10*, *IL12 (p70)*, *IL13*, *IL17*, *CXCR1*, *CXCR2*, *PTGS2*, *PTGES2*, *NFKB1*, and *TNFα*) [30,33,34,35,36,37,38,39,40,41,42]; and the fourth group consists of tumor suppressor genes including *P53*, *BRCA1* [43], *hMLH1*, *hMSH2*, *hMSH6*, and hPMS2 [44,45] (Table 1). 

**Figure 1:**
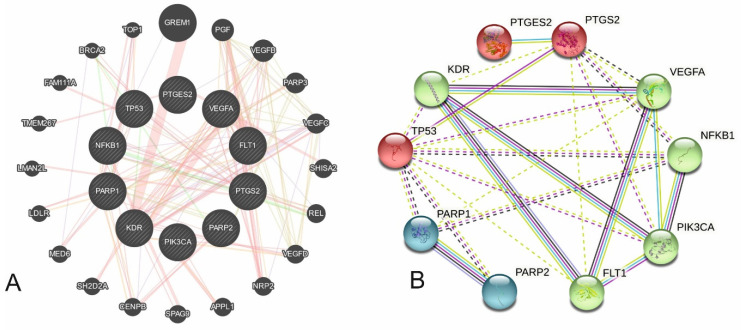
(**A**) Gene–gene interaction of the 11 main genes that explain how aspirin can improve cancer survival (grey shaded nodes are primary input genes, grey non-shaded nodes are the secondary genes showing interaction with primary genes) (**B**). Protein–protein interaction of 11 proteins synthesized by the 11 main genes identified, showing three clusters of proteins; the first cluster is co-expression of PTGS2, PTGES2, and P53 (red), while the second cluster is of cell cycle regulators (green), and the third is of DNA repair genes (blue).

Of these 41 genes, 11 (*PTGS2*, *PIK3CA*, *PARP1*, *PARP2*, *VEGFA*, *KDR*, *PTGES2*, *NFKB1*, *P53*, *FLT1*, *VEGFR*) had mechanisms that were directly regulated by aspirin or interacted with aspirin (Figure 1, Supplementary File S1). The remaining 30 genes had indirect involvement or acted through regulation of one of these 11 genes or inflammatory pathways. Focusing on the 11 genes with direct aspirin regulation or interaction formed the basis of the main analysis. The inflammatory pathway was used for secondary analysis. After categorizing the genes into the above four groups, GeneMANIA identified 34 co-expression, 2 genetic, 54 physical, and 23 predicted interactions between these 11 genes (Figure 1A, Supplementary File S1). A total of 44 shared protein domains were identified among the interacting genes. Protein–protein interactions occurred in three clusters of three, five, and two proteins (Figure 1B). PTGES2 only interacted with PTGS2. All other proteins interacted with each other, except PARP2, which had no interaction with the FLT protein. This analysis confirmed that *PTGS2* and *PTGES2* are the central genes through which aspirin impacts cancer survival. 

**Figure 2:**
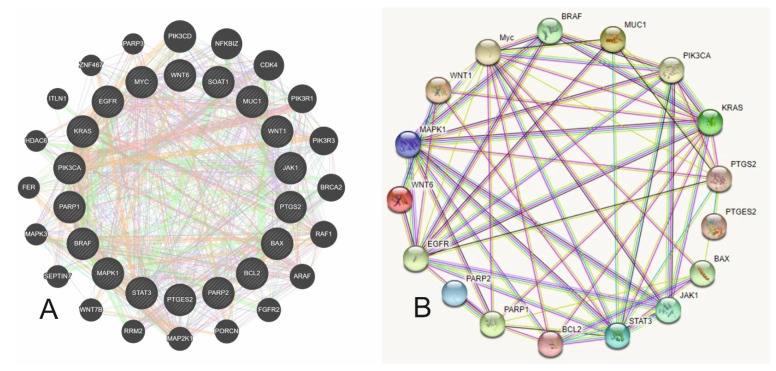
(**A**). Showing gene–gene interactions of PTGS2/PTGES2 with cell cycle regulators and cellular proliferation genes identified by data and text mining (purple—co-expression, pink—physical interaction, green—genetic interaction, orange—predicted interactions) (**B**). The protein–protein network of PTGS2/PTGES2 with cell cycle regulators and cellular proliferation proteins has 18 nodes and 62 edges. The interactions are statistically significant (*p* = 2.75 × 10^−15^) (light blue—curated databases, pink—experimentally determined; green—predicted gene neighborhood; red—predicted gene fusion; blue—predicted gene co-occurrence).

Given this confirmation that *PTGS2* and *PTGES2* are the primary genes inhibited by aspirin, we evaluated how these two primary genes interact with the other 39 genes, which we categorized into three groups based on their function, i.e., cell signaling and cell proliferation, cytokines and interleukins, and tumor suppressor genes. The analysis of *PTGS2* and *PTGES2* with genes participating in cell signaling and proliferation showed 37% co-expression and 20% physical interaction with 18 primary nodes and 20 secondary nodes, reflecting how these genes relate to one another (Figure 2A). The protein–protein network of these three groups of genes had 18 nodes and 62 edges (Figure 2B). The p value of the network was highly significant (*p* = 2.75 × 10^−15^), suggesting significantly more interactions than expected. The highest number of interactions with PTGS2 was seen with the MAP kinase and EGFR pathways, suggesting that COX2 (PTGS2) suppression may lead to inhibition of MAP kinase and EGFR pathways and hence a reduction in cellular growth and proliferation.

We then examined the interaction of *PTGS2* and *PTGES2* with the interleukin and cytokine genes. The gene–gene network showed a high level of co-expression of these genes (85%), which was expected as the *COX2* gene, and prostaglandin controls the synthesis of interleukins and cytokines (Supplementary Figure S1A). The protein–protein network of these genes was equally rich, with 18 nodes and 122 edges (Supplementary Figure S1B). The PPI enrichment value was highly statistically significant (*p* ≤ 1.0 × 10^−16^), indicating that there were many more interactions than expected by chance.

The network of *PTGS2* and *PTGES2* with tumor suppressor genes showed nearly 70% physical interactions with 21 secondary nodes between these genes (Supplementary Figure S2A). While the protein–protein interaction network (Supplementary Figure S2B) had five nodes and five edges, the interaction was not statistically significant (*p* = 0.07), suggesting that COX2 does not directly regulate these tumor suppressor genes. In contrast, the cell signaling and proliferation proteins (Supplementary Figure S2C) and the cytokine and interleukin gene proteins (Supplementary Figure S2D) did interact with the tumor suppressor gene proteins (*p* ≤ 1.0 × 10^−16^), suggesting that aspirin indirectly regulates tumor suppressor genes through its impact on cell signaling and the proliferation gene and interleukin and cytokine gene expression. All gene–gene interactions between the four groups (primary genes, cell signaling and cell proliferation genes, cytokine and interleukin genes, and tumor suppressor genes) are detailed in Supplementary File S2.

**Figure 3:**
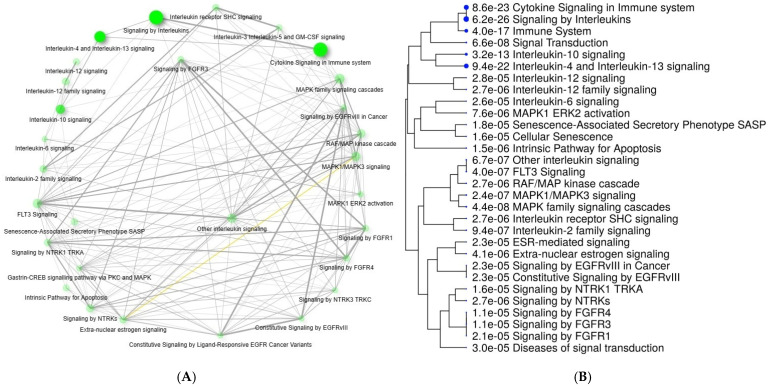
(**A**) Pathway enrichment with crosstalk between identified pathways. (**B**) Tree analysis of the pathway based on the *p* value for each pathway on enrichment. Pathways with many shared genes are clustered together. Bigger dots indicate more significant *p*-values.

**Figure 4:**
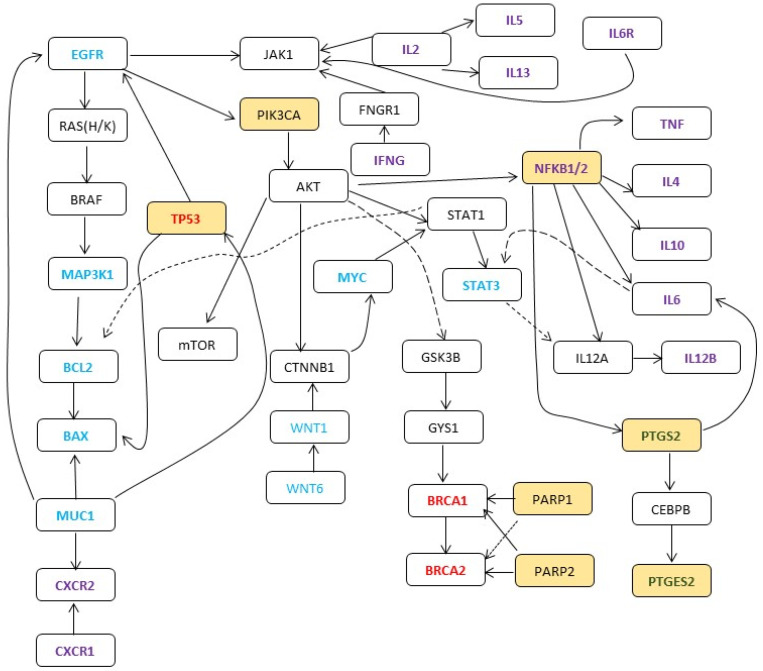
Proposed mechanism of aspirin in inhibiting cellular proliferation and increasing in autophagy, DNA repair, and apoptosis through complex crosstalk. Genes identified through data mining are in shaded boxes. Primary genes are in green text, oncogenes and cell cycle regulator genes in blue text, tumor suppressor genes in red text, cytokines and interleukins in purple text, and secondary genes are in black text. Dotted line show predicted interaction.

A pathway analysis with Reactome showed that the largest number of genes interacted with interleukin (Supplementary File S3) and signal transduction pathways (Supplementary File S4). Additional interactions were identified in DNA repair, autophagy, extracellular matrix, and apoptosis pathways (Supplementary Files S5–S8). Based on these pathways, a pathway diagram was created that incorporated most genes and pathways (Figure 3A,B). The crosstalk between the genes and the pathways is shown in Figure 4.

## 4. Discussion

There are many studies on cancer prevention using aspirin, and its effect, along with that of other COX2 inhibitors, is well known. The results of the randomized controlled trials have been mixed, and the findings from the low-dose trial and subsequent non-randomized observational studies suggest that the addition of aspirin to the standard of care improves cancer survival. However, the mechanism by which aspirin improves survival is not clear. This study is the first to our knowledge to comprehensively evaluate the literature and apply bioinformatics to investigate the underlying mechanism.

Our literature search identified 41 genes that are related to aspirin and cancer survival and that fell into four clusters, including the primary genes directly regulated by *PTGS2* and *PTGES2,* oncogenes and cell cycle regulators (16 genes), interleukin and cytokines regulators (16 genes), and tumor suppressor genes (7 genes). Of the 11 main genes that are regulated by aspirin or interact with aspirin, the DNA repair pathway, especially the homologous recombination (HR) pathway, showed co-expression of *PTGS2* with *TP53*, *PARP1*, and *PARP2*. 

Several previous studies have shown that aspirin can affect DNA repair of genes. For example, treatment of a DNA MMR competent/p53 mutant colorectal cancer cell line with aspirin for 48 h led to DNA damage pathway gene expression, including *BRCA1* [45]. A later study found that feeding aspirin to Dalton cell lymphoma-bearing mice resulted in cell cycle arrest in the G0/G1 phase [34]. In addition, aspirin was found to lower the number of somatic mutations, including mutations in *TP53* in Barret’s esophagus patients [40]. The potential of altering the levels of NFκB and its use as a biomarker are being tested in ASAMET (NCT03047837) trial, wherein both aspirin and metformin are given to patients with stage I-III colorectal patients, and the results are awaited [46]. 

Despite the availability of cell line and animal model data, it is still not clear why there is co-expression of *COX2* (*PTGS2*) with DNA repair pathway genes. Rationally, as COX2 increases cellular proliferation, it should inhibit DNA repair and apoptosis proteins, as is seen with counter-regulation of the BCL2/BAX/Caspase cascade. Two possible mechanisms are through the regulation of *MYC* and *MUC1*. Alternatively, the co-expression of COX2 and DNA repair pathway genes could be due to an increase in DNA synthesis, thereby increasing the DNA repair cascade to check the DNA that is being newly synthesized. We demonstrate the regulation of DNA repair genes through crosstalk of the interleukin pathway that regulates *JAK1*, *PI3KC,* and *AKT*, which in turn regulate the p53 pathway and another interaction through CXCR regulation through Muc1 (Figure 4). This hypothesis of complex crosstalk might be more plausible, but the exact mechanisms involved are not clearly understood. Additional studies are required to understand this process fully.

The effect of *PTGS2/PTGES2* on interleukin pathway activation is more obvious and can be explained by its effect on arachidonic acid metabolism and increased synthesis of interleukins and cytokines. Similarly, the effects on cellular proliferation and signal transduction pathways are also explainable through direct [45] or interleukin mechanisms [23,33,34,47]. 

Though most of the RCTs failed to demonstrate any survival benefit from the addition of aspirin to the standard of care, the benefit is seen in one RCT of low-dose aspirin, in observational studies, and in a meta-analysis that included these observational studies. This inconsistency could be due to the use of different doses of aspirin or inherent biases in the observational studies like adherence bias, healthy user bias, etc. Further, most earlier studies were based on the effect of aspirin on the blockade of the COX2 or thromboxane (Tx) pathway and assumed this blockade to be the main mechanism through which aspirin exerts its benefit on cancer survival. It is well known that at a low dose, aspirin irreversibly acetylates serine 530 of cyclooxygenase (COX)-1. This inhibits platelets from generating thromboxane A2, thus resulting in an antithrombotic effect by making TBX2A unavailable to bind TBXA2R. However, how this effect reduces cancer survival is not known. The present study is the first to demonstrate that aspirin can improve survival by an interplay of 41 genes, 2 of which (PTGS2 (COX2) and PTGES2) are the primary target of aspirin. The effect is mediated through six primary pathways: the interleukin, extracellular matrix, signal transduction, apoptosis, autophagy, and DNA repair pathways. 

## 5. Conclusions

This study demonstrates that aspirin’s regulation of cancer is more complex than previously understood. However, as this is a bioinformatic analysis of published research, it has inherent shortcomings, as the study results are not validated by experimental studies. More randomized controlled trials evaluating the benefit of adding aspirin on cancer survival and incorporating molecular studies assessing interleukins and cytokines are required to fully understand the mechanisms by which aspirin appears to improve survival in cancer patients. 

## Figures and Tables

**Table 1:** The genes identified as effectors to increase survival in patients receiving Aspirin, their grouping according to the function.

Group	Gene	Function
1 Primary genes	Prostaglandin-endoperoxide synthase 2 (prostaglandin G/H synthase and cyclooxygenase) PTGS2/COX2	Synthesis of enzyme cyclooxygenase 2 (COX 2) that converts arachidonic acid to prostaglandin endoperoxide H2
	PTGES2—prostaglandin E synthase 2	Encodes membrane-associated prostaglandin E synthase, which catalyzes the conversion of prostaglandin H2 to prostaglandin E2; also activates gamma-interferon-activated transcription element (GATE)
2 Oncogenes and cell cycle regulators	MYC—MYC proto-oncogene, bHLH transcription factor	Encodes a nuclear phosphoprotein that plays a role in cell cycle progression, apoptosis, and cellular transformation
	EGFR—epidermal growth factor receptor	Encodes for epidermal growth factor receptor protein that regulates epithelial tissue development and homeostasis
	BCL2—B cell lymphoma 2	Encodes protein that regulates apoptosis. Increases cellular survival by inhibiting proapoptotic proteins
	WNT1—WNT family member 1; Proto-oncogene Int-1 homolog	Encodes secreted signaling proteins that increase cell growth and division
	KRAS—Kirsten rat sarcoma viral oncogene homolog	Encodes tumor oncogene Kras protein part of RAS/MAPK pathway among other signal transduction pathways
	WNT6—Wingless-type MMTV integration site family, member 6	Highly conserved gene, encodes wnt6 protein that regulates cell growth and differentiation through wnt pathway
	BRAF—raf murine sarcoma viral oncogene homolog B	Member of raf kinase family, controls cell proliferation by regulating signal transduction protein kinases
	MUC1—Mucin 1, cell surface-associated; polymorphic epithelial mucin (PEM); epithelial membrane antigen (EMA)	Protective function as it binds to foreign pathogens, also regulates cell signaling
	PIK3CA—Phosphatidylinositol-4,5-Bisphosphate 3-Kinase Catalytic Subunit Alpha	Encodes oncogenic protein that catalyzes ATP to phosphorylate PtdIns, PtdIns4P, and PtdIns(4,5)P2
	PARP1—Poly[ADP-ribose] polymerase 1 (PARP-1); NAD+ ADP-ribosyltransferase 1 or poly[ADP-ribose] synthase 1	ADP-ribosylation, repair of single-strand breaks, and with BRCA, double-stranded breaks
	PARP2—Poly [ADP-ribose] polymerase 2	Encodes poly(ADP-ribosyl)transferase-like 2 protein, a catalytic domain capable of catalyzing a poly(ADP-ribosyl)ation reaction
	STAT3—Signal transducer and activator of transcription 3	Transcription activator after being phosphorylated by receptor-associated Janus kinases (JAK)
	JAK1—Janus kinase 1	Tyrosine kinase protein essential for signaling type I and type II cytokines. Promotes cell division
	MAPK1—Mitogen-activated protein kinase 1, also known as ERK2	Member of the MAP kinase family regulating extracellular signal-regulated kinases
	STAT—Signal transducer and activator of transcription	Intracellular transcription factors that mediate cellular immunity, proliferation, apoptosis, and differentiation
	BAX—bcl-2-like protein 4	Forms heterodimers with BCL2 and regulates apoptosis. It is a proapoptotic factor that stimulates release of caspases
Group 3—cytokines and Interleukins	IFNG—Interferon gamma	Class II interferon, activator of macrophages and inducer of major histocompatibility complex class II molecule expression
	IL1B—Interleukin 1Beta, also known as leukocytic pyrogen, leukocytic endogenous mediator, mononuclear cell factor, lymphocyte activating factor	Interleukin 1 family of cytokines, released from macrophages, activated by caspase; it mediates inflammatory response and other cellular activities, including cell proliferation, differentiation, and apoptosis
	IL2—Interleukin 2	Improves tolerance and immunity, primarily via its direct effects on T cells. Improves cell killing by NK cells and activated T cells
	IL4—Interleukin 4	Induces differentiation of naive helper T cells (Th0 cells) to Th2 cells
	IL5—Interleukin 5	Stimulates B cell growth and increases immunoglobulin (IgA) secretion
	IL6—Interleukin 6	Proinflammatory cytokine secreted by macrophage and an anti-inflammatory myokine. It binds to pattern recognition receptors (PRRs), including Toll-like receptors (TLRs)
	IL7—Interleukin 7	Hematopoietic growth factor secreted by stromal cells in the bone marrow and thymus. Participates in proliferation during certain stages of B-cell maturation, T and NK cell survival, development, and homeostasis
	IL8—Interleukin 8	Chemokine produced by macrophages and epithelial cells. Induces chemotaxis and angiogenesis
	IL10—Interleukin10; human cytokine synthesis inhibitory factor (CSIF)	Primarily produced by monocytes, participates in immunoregulation and inflammation. Blocks NF-κB, regulates JAK-STAT pathway
	IL12B—Interleukin 12 subunit beta; (natural killer cell stimulatory factor 2, cytotoxic lymphocyte maturation factor p40, or interleukin-12 subunit p40)	Secreted by activated macrophages, inducer of Th1 cell development.
	IL13—Interleukin 13	Functions similar to IL3. Induces a class of protein-degrading enzymes, known as matrix metalloproteinases (MMPs)
	IL17—interleukin 17	Immune regulatory cytokine, induces and mediates proinflammatory response. Induces expression of keratinocytes
	CXCR1—C-X-C motif chemokine receptor 1; Interleukin 8 receptor, alpha or CD181	G-protein-coupled receptor family that binds to IL8 and increases cellular proliferation
	CXCR2—C-X-C motif chemokine receptor 2; Interleukin 8 receptor, beta	Binds with IL8 and transduces the signal through a G-protein-activated second messenger system. Mediates angiogenic effect of IL8 on endothelial cells
	NFκB—Nuclear factor kappa-light-chain-enhancer of activated B cells	A primary transcription factor and regulator of signaling response, suppresses TNF, induces cytotoxicity, regulates TRAF1 and TRAF2
	TNF—Tumor necrosis factor (cachexin, or cachectin; also called as tumor necrosis factor alpha or TNF-α)	Adipokine and a cytokine, binds to two receptors, TNFR1 and 2. Regulates NFκB and TRAF1 and 2, activates MAP kinase pathway, cell differentiation, and proliferation
4—Tumor suppressor genes	TP53—tumor protein 53; transformation-related protein 53 (TRP53)	Tumor suppressor gene, DNA repair, cell cycle arrest in G1/S phase, initiator of apoptosis, senescence response to short telomeres
	BRCA1—Breast cancer type 1 susceptibility gene	Repair of double-stranded DNA breaks; mismatch repair
	BRCA2—breast cancer type 2 susceptibility gene	Encodes BRCA2 protein with functions similar to that of BRCA1. Forms BRCA1-PALB2-BRCA2 complex
	MLH1—mutL homolog 1	DNA mismatch repair of genes
	PMS2—PMS1 homolog 2	DNA mismatch repair of genes
	MSH2—mutS homolog 2	Microsatellite instability-associated gene altered in microsatellite sequences (RER+ phenotype) in HNPCC
	MSH6—mutS homolog 6	DNA mismatch repair; forms MSH recognition complex with MSH2

## Data Availability

All the data are submitted as supplementary files or included in the manuscript.

## Supplementary Materials

The following supporting information can be downloaded at https://www.mdpi.com/article/10.3390/cancers16010223/s1: Supplementary File S1: Table showing gene–gene interactions between 11 main genes. Supplementary File S2: Excel file table showing gene–gene interactions between all four group of genes. Supplementary File S3: Interaction of interleukin pathway with 37 identified genes, produced by Reactome database. Supplementary File S4: Interaction of signal transduction pathway with 37 identified genes, produced by Reactome database. Supplementary File S5: Interaction of apoptosis pathway with 37 identified genes, produced by Reactome database. Supplementary File S6: Interaction of extracellular matrix pathway with 37 identified genes, produced by Reactome database. Supplementary File S7: Interaction of DNA repair pathway with 37 identified genes, produced by Reactome database. Supplementary File S8: Interaction of autophagy pathway with 37 identified genes, produced by Reactome database. Figure S1: (A). Gene-gene interaction of PTGS2/PTGES2 with Interleukin and cytokine pathway genes (purple—co-expression, pink—physical interaction, green—genetic interaction, orange—predicted interactions). (B). The statistically significant (*p* ≤ 1.0e^−16^), protein-protein network of PTGS2/PTGES2 with interleukin and cytokine pathway proteins (light blue—curated databases, pink—experimentally determined; green—predicted gene neighborhood; red—predicted gene fusion; blue—predicted gene co-occurrence). Figure S2: (A). The gene-gene interaction of PTGS2 and PTGES2 with tumor suppressor genes (purple—co-expression, pink—physical interaction, green—genetic interaction, orange—predicted interactions). (B). protein-protein interaction of PTGS2/PTGES2 with tumor suppressor proteins having 5 nodes and 5 edges, the interaction was statistically not significant (*p* = 0.07). (light blue—curated databases, pink—experimentally determined; green—predicted gene neighborhood; red—predicted gene fusion; blue—predicted gene co-occurrence). (C) Protein-protein interaction of Cellular proliferation and tumor oncogenes with tumor suppressor genes showing statistically significant interactions (*p* ≤ 1.0e^−16^). (light blue—curated databases, pink—experimentally determined; green—predicted gene neighborhood; red-predicted gene fusion; blue-predicted gene co-occurrence). (D) Protein-protein interaction of interleukin and cytokine genes with tumor suppressor genes showing statistically significant interactions (*p* ≤ 1.0e^−16^). (light blue—curated databases, pink—experimentally determined; green—predicted gene neighborhood; red—predicted gene fusion; blue—predicted gene co-occurrence).

## Abbreviations

COX	cyclooxigenase
PTGS	prostaglandin-endoperoxide synthase
PTGES	prostaglandin E synthase
EGFR	epidermal growth factor receptor
JAK	Janus kinase
BRCA	BReast CAncer gene
PARP	Poly (ADP-ribose) polymerases
DNA	Deoxyribonucleic acid
RCTs	randomized controlled trials
HR	hazard ratio
MMR	mismatch repair
MeSH	Medical Subject heading
ASAMET	Aspirin and Metformin in Stage I–III Colorectal Cancer Patients
