# Supplementary material for: Aspirin and Cancer Survival: An Analysis of Molecular Mechanisms
Source: Cancers (Basel). 2024 Jan 3;16(1):223. doi: 10.3390/cancers16010223 (PMC10778469; doi:10.3390/cancers16010223)
Supplement: Supplementary file 1 [file cancers-16-00223-s001.zip › Additional File S1.pdf]

Additional file S1: Showing type of gene-gene interaction and weight between 11 main and their interacting genes

Gene 1	Gene 2	Weight	Network group
VEGFA	FLT1	0.020744	Co-expression
VEGFR	FLT1	0.016419	Co-expression
p53	NFKB1	0.010398	Co-expression
GREM1	NFKB1	0.012934	Co-expression
VEGFC	FLT1	0.015285	Co-expression
VEGFC	VEGFR	0.013902	Co-expression
SHISA2	VEGFC	0.013704	Co-expression
NFKB1	PTGS2	0.025296	Co-expression
NFKB1	PARP1	0.02428	Co-expression
HIF1A	VEGFA	0.013041	Co-expression
HRAS	CENPB	0.010719	Co-expression
VEGFR	FLT1	0.01931	Co-expression
VEGFB	PTGES2	0.009474	Co-expression
VEGFR	FLT1	0.014055	Co-expression
VEGFC	VEGFR	0.005695	Co-expression
NRP2	VEGFC	0.010776	Co-expression
HIF1A	REL	0.010267	Co-expression
NRP1	VEGFR	0.013921	Co-expression
CENPB	VEGFB	0.005867	Co-expression
p53	PTGS2	0.009865	Co-expression
VEGFC	VEGFR	0.011381	Co-expression
VEGFC	PGF	0.010706	Co-expression
REL	VEGFA	0.020182	Co-expression
HIF1A	VEGFR	0.009953	Co-expression
HRAS	VEGFB	0.012284	Co-expression
VEGFA	PTGS2	0.010792	Co-expression
VEGFC	NFKB1	0.015597	Co-expression
FLT4	VEGFR	0.015038	Co-expression
HIF1A	PTGS2	0.011642	Co-expression
NRP1	PTK2	0.012855	Co-expression
HRAS	VEGFB	0.022873	Co-expression
NFKB1	PTGS2	0.008028	Co-expression
VEGFC	VEGFR	0.002712	Co-expression
REL	NFKB1	0.008002	Co-expression
ARNT	PARP1	0.011595	Co-expression
CENPB	VEGFR	0.002159	Co-expression
p53	VEGFR	0.251096	Genetic Interactions
REL	NFKB1	1	Genetic Interactions
VEGFA	FLT1	0.765367	Physical Interactions
VEGFR	VEGFA	0.414214	Physical Interactions
GREM1	VEGFR	0.765367	Physical Interactions

VEGFA	FLT1	0.259808	Physical Interactions
VEGFR	VEGFA	0.294428	Physical Interactions
p53	PTGS2	0.174421	Physical Interactions
PGF	FLT1	0.353001	Physical Interactions
VEGFC	VEGFR	0.361476	Physical Interactions
FLT4	VEGFC	0.696859	Physical Interactions
NRP2	VEGFA	0.335654	Physical Interactions
NRP2	PGF	0.456053	Physical Interactions
PLCG1	FLT1	0.080979	Physical Interactions
PLCG1	VEGFR	0.09177	Physical Interactions
HIF1A	p53	0.015081	Physical Interactions
ARNT	HIF1A	0.068108	Physical Interactions
SPAG9	NFKB1	0.58416	Physical Interactions
APPL1	PIK3CA	0.554	Physical Interactions
HRAS	PIK3CA	0.222599	Physical Interactions
VEGFA	FLT1	0.6867	Physical Interactions
VEGFB	VEGFA	0.352144	Physical Interactions
REL	NFKB1	0.086294	Physical Interactions
ARNT	HIF1A	0.077247	Physical Interactions
SHISA2	PTGES2	0.527581	Physical Interactions
VEGFR	VEGFA	0.344082	Physical Interactions
p53	PTGS2	0.008964	Physical Interactions
p53	PARP1	0.004882	Physical Interactions
NOS3	p53	0.023252	Physical Interactions
HIF1A	p53	0.003357	Physical Interactions
PTK2	p53	0.003493	Physical Interactions
NRP1	PGF	0.870522	Physical Interactions
CENPB	PARP2	0.539726	Physical Interactions
CENPB	PARP1	0.068953	Physical Interactions
SPAG9	NFKB1	0.098695	Physical Interactions
p53	PTGS2	0.098602	Physical Interactions
p53	PARP1	0.012737	Physical Interactions
CENPB	PARP2	0.556068	Physical Interactions
CENPB	PARP1	0.170838	Physical Interactions
VEGFR	VEGFA	0.164772	Physical Interactions
p53	PARP1	0.006442	Physical Interactions
FLT4	VEGFR	0.169992	Physical Interactions
PLCG1	VEGFR	0.024351	Physical Interactions
PTK2	p53	0.008655	Physical Interactions
NRP1	VEGFA	0.656783	Physical Interactions
NRP1	VEGFR	0.120273	Physical Interactions
HRAS	PIK3CA	0.032061	Physical Interactions
VEGFA	FLT1	0.247389	Physical Interactions
VEGFR	VEGFA	0.093198	Physical Interactions
VEGFB	FLT1	0.842676	Physical Interactions
FLT4	VEGFC	0.519377	Physical Interactions

VEGFD	FLT4	0.519377	Physical Interactions
PARP3	PARP1	0.290457	Physical Interactions
NRP1	VEGFA	0.259738	Physical Interactions
ARNT	HIF1A	0.069681	Physical Interactions
APPL1	PIK3CA	0.135466	Physical Interactions
VEGFR	VEGFA	0.360202	Predicted
FLT4	VEGFC	0.578164	Predicted
VEGFD	VEGFR	0.63931	Predicted
PLCG1	FLT1	0.157518	Predicted
CENPB	PARP1	0.52775	Predicted
PARP1	PARP2	0.121717	Predicted
NFKB1	PARP1	0.017072	Predicted
p53	PARP1	0.006247	Predicted
PGF	VEGFA	0.153457	Predicted
VEGFB	VEGFA	0.21321	Predicted
FLT4	FLT1	0.211842	Predicted
VEGFD	VEGFC	0.796225	Predicted
VEGFD	VEGFB	0.380021	Predicted
NRP2	PGF	0.140402	Predicted
PARP3	PARP1	0.335989	Predicted
PTK2	FLT1	0.03153	Predicted
PTK2	p53	0.004455	Predicted
PTK2	FLT4	0.075653	Predicted
PTK2	PLCG1	0.011178	Predicted
NRP1	PGF	0.103095	Predicted
NRP1	NRP2	0.086735	Predicted
CENPB	PARP1	0.335989	Predicted
APPL1	PIK3CA	0.127628	Predicted
PARP1	PARP2	0.152838	Shared protein domains
VEGFR	FLT1	0.027243	Shared protein domains
p53	NFKB1	0.009621	Shared protein domains
PGF	VEGFA	0.052649	Shared protein domains
VEGFC	VEGFA	0.055282	Shared protein domains
VEGFC	PGF	0.053835	Shared protein domains
VEGFB	VEGFA	0.083625	Shared protein domains
VEGFB	PGF	0.052649	Shared protein domains
VEGFB	VEGFC	0.055282	Shared protein domains
FLT4	FLT1	0.031181	Shared protein domains
FLT4	VEGFR	0.03434	Shared protein domains
VEGFD	VEGFA	0.054009	Shared protein domains
VEGFD	PGF	0.052595	Shared protein domains
VEGFD	VEGFC	0.055226	Shared protein domains
VEGFD	VEGFB	0.054009	Shared protein domains
REL	NFKB1	0.054156	Shared protein domains
REL	p53	0.013134	Shared protein domains
PARP3	PARP2	0.277026	Shared protein domains

PARP3	PARP1	0.152838	Shared protein domains
PTK2	PIK3CA	0.002971	Shared protein domains
PTK2	FLT1	0.006622	Shared protein domains
PTK2	VEGFR	0.007295	Shared protein domains
PTK2	FLT4	0.008728	Shared protein domains
NRP1	NRP2	0.075612	Shared protein domains
ARNT	HIF1A	0.015578	Shared protein domains
PARP1	PARP2	0.13543	Shared protein domains
VEGFR	FLT1	0.072263	Shared protein domains
PGF	VEGFA	0.14711	Shared protein domains
VEGFC	VEGFA	0.14711	Shared protein domains
VEGFC	PGF	0.14711	Shared protein domains
VEGFB	VEGFA	0.14711	Shared protein domains
VEGFB	PGF	0.14711	Shared protein domains
VEGFB	VEGFC	0.14711	Shared protein domains
FLT4	FLT1	0.070847	Shared protein domains
FLT4	VEGFR	0.070847	Shared protein domains
VEGFD	VEGFA	0.14711	Shared protein domains
VEGFD	PGF	0.14711	Shared protein domains
VEGFD	VEGFC	0.14711	Shared protein domains
VEGFD	VEGFB	0.14711	Shared protein domains
REL	NFKB1	0.046424	Shared protein domains
PARP3	PARP2	0.215495	Shared protein domains
PARP3	PARP1	0.13543	Shared protein domains
NRP1	NRP2	0.059382	Shared protein domains
ARNT	HIF1A	0.019222	Shared protein domains
