# Supplementary material for: Aspirin and Cancer Survival: An Analysis of Molecular Mechanisms
Source: Cancers (Basel). 2024 Jan 3;16(1):223. doi: 10.3390/cancers16010223 (PMC10778469; doi:10.3390/cancers16010223)
Supplement: Supplementary file 1 [file cancers-16-00223-s001.zip › Additional File S3.pdf]

Additional file S3: Interaction of interleukin pathway with 37 identified genes produced by Reactome database

Input	UniProt Id	Input	UniProt Id	Input	UniProt Id
BCL2	P10415	IFNG	P01579	IL10	P22301
IL12B	P29460	IL13	P35225	IL17	Q16552
IL1B	P01583, P01584, Q8WWZ1	IL2	P60568	IL4	P05112
IL5	P05113	IL6	P05231	IL7	P13232
IL8	P10145	JAK1	P23458	JAK2	O60674
JAK3	P52333	MAPK	P28482	MUC1	P15941
Myc	P01106	NFKB1	P19838	PIK3CA	P42336
PTGS2	P35354	STAT	P40763	STAT3	P40763
TNF	P01375	p53	P04637		
Input	Ensembl Id	Input	Ensembl id	Input	Ensembl id
BCL2	ENSG00000171791	IFNG	ENSG00000111537	IL10	ENSG00000136634
IL12B	ENSG00000113302	IL17	ENSG00000112115	IL1B	ENSG00000125538
IL6	ENSG00000136244	IL8	ENSG00000169429	MUC1	ENSG00000185499
Myc	ENSG00000136997	PTGS2	ENSG00000073756	TNF	ENSG00000232810
p53	ENSG00000141510				

Interactors found in the analysis (22)

Input	UniProt Id	Interacts with	Input	UniProt Id	Interacts with
BCL2	P10415	P00441	BRAF	P15056	P63104, P38646
EGFR	P00533	P29353, P22681, P14625, Q13905, Q06124, P38646, Q9UQC2, P15498, P42224, O00170, P62993, P14174, Q9Y6K9, P23528, P45983, O14543, P63104, P07355, P40763	IL10	P22301	Q08334
IL12B	P29460	P29460, Q9NPF7, P29459	IL13	P35225	P78552, Q14627
IL2	P60568	P01589, P14784	IL4	P05112	P24394, P78552, P31785
IL5	P05113	Q01344, P32927	IL6	P05231	P40189, P08887
IL7	P13232	P16871	JAK1	P23458	P42224, O60674, P40763
JAK2	O60674, Q62120	Q01344, Q5VWK5, O60674, P32927, P23458	JAK3	P52333	P17987
Myc	P01106, EBI-1265559	O15111, Q9Y6K9, P23771, P40763	NFKB1	P25799-1, P19838, P19838-1	P41279, O15111, P19838, Q9Y6K9, Q8NFZ5, O14920, P25963
PARP1	P09874	Q13007	PIK3CA	P42336	P62993, P01100
STAT	P35610, EBI-10097350, EBI-10952519	P42224, Q14765, P27824, P40763	STAT3	EBI-9914958, P42227, P40763	P42224, P40189, P43405, P62993, P22681, P23458, P49137, Q99062, P40763
TNF	P01375	Q16623, Q9Y6K9	p53	P04637	P17987, O14920, P63104, P38646
