# Supplementary material for: Aspirin and Cancer Survival: An Analysis of Molecular Mechanisms
Source: Cancers (Basel). 2024 Jan 3;16(1):223. doi: 10.3390/cancers16010223 (PMC10778469; doi:10.3390/cancers16010223)
Supplement: Supplementary file 1 [file cancers-16-00223-s001.zip › Additional File S4.pdf]

Additional file S4: Interaction of signal transduction pathway with 37 identified genes produced by Reactome database

24 submitted entities found in this pathway, mapping to 31 Reactome entities

Input	UniProt Id	Input	UniProt Id	Input	UniProt Id
BAX	Q07812	BCL2	P10415	BRAF	P15056
CXCR1	P25024	CXCR2	P25024, P25025	EGFR	P00533, P04626
IL2	P60568	IL5	P05113	IL6	P05231
IL8	P10145	JAK1	P23458	JAK2	O60674
JAK3	P52333	KRAS	P01116, P01116-1, P01116-2	MAPK	P28482
Myc	P01106	NFKB1	P19838	PARP1	P09874
PIK3CA	P42336	STAT3	P40763	TNF	P01375
WNT1	P04628	WNT6	Q9Y6F9	p53	P04637

Input	Ensembl Id	Input	Ensembl Id
BCL2	ENSG00000171791	Myc	ENSG00000136997, ENST00000377970

Interactors found in the analysis (24)

Input	UniProt Id	Interacts with	Input	UniProt Id	Interacts with
BAX	Q07812, Q07813-1	Q07817, P10415, O43521, Q07812	BCL2	P10415	P22736, Q92934, P10415, O43521, P28482, Q07812
BRAF	P15056	P04049, P36507, P15056, P31946, Q02750, P63104	BRCA1	P38398	P24385, Q8WX92, P52292, P62136, Q7Z569
BRCA2	P51587	P09874	CXCR2	P25025	P10145
EGFR	P00533-4, P00533	P25098, Q14247, P35813, P07900, P19174, P12931, P15498, Q05209, P46109, Q99962, Q99963, Q96JA1, O14544, Q13480, O14543, P27986, P46108, P40763, P04049, Q13322, Q68CZ2, P46934, P16234, Q99952, Q05513, P41240, P00533, P29353, P22681, P30307, P32121, P29350, Q06124, Q03135, Q99075, Q05397, P08581, P17252, P23528, Q07889, Q9UQB8, Q99704, P63104, P01135, P42685, P01133, Q9UGK3, P31946, Q14956, Q12933, P18031, P31749, Q13905, P52306, P30530, Q02790, Q9UQC2, Q9UNE7, P00519, P53041, P06493, Q16620, P45983, P04792, Q13153	IL10	P22301	P25490
IL2	IL2	Q13547	IL4	P05112	P31785
IL8	EBI-1566585, P10145	Q92793, P25025	JAK1	P23458	O60674, P40763
JAK2	O60674, Q62120	P46527, O60674, P18031	JAK3	P52333	Q9UNE7
KRAS	P01116, P01116-2, P32883	P04049, P15056, P01116-2, P61586	MUC1	P15941-11, P15941	P01350, P00519, P12931, P00533
Myc	P01106, EBI-1265559	Q8N6T7, O15111, P06307, Q13526, Q13547, Q14839, O43524, Q9Y4A5, P37173, P52292, P08047, O60341, O15169, P49841, P04792, P23771, P40763	NFKB1	P25799-1, P19838-PRO_0000030311, P19838, P25799	Q15788, O15111, Q8IZL8, Q13547, O00255, Q9Y297, P35222, P25963
PARP1	P09874	P22415, Q9NTX7, P09874	PIK3CA	P42336	P27986, P35568, P42336, P01100
STAT	EBI-10952519	P40763	STAT3	EBI-9914958, P40763-2, P40763	P22681, P18031, Q9NP31, Q9UBE8, P12931, O43318, P22736, P43405, Q9NWQ8, P08047, P49137, P00533, P06401, P40763
TNF	P01375	Q15628, O15552, Q12933, Q13546, P01375	p53	P04637, P04637-7, P04637-1	Q09472, Q9Y265, P10415, P29590, Q05397, P22736, P17844, Q92793, O14641, P08047, Q92993, P63104, Q93009, P09874, P49757, Q13526, Q13547, O14980, Q15648, O43524, Q9UHC7, Q06330, Q96ST3, Q00987, P35232, Q15291, P04271, O15169, P49841, P04792, Q15796, Q9UBL3

10 submitted entities found in this pathway, mapping to 12 Reactome entities

Input	UniProt Id	Input	UniProt Id	Input	UniProt Id
BAX	Q07812	BRAF	P15056	EGFR	P00533, P04626
JAK2	O60674	JAK3	P52333	KRAS	P01116-1, P01116-2
MAPK	P28482	Myc	P01106	PIK3CA	P42336
STAT3	P40763				

Interactors found in the analysis (16)

Input	UniProt Id	Interacts with	Input	UniProt Id	Interacts with
BAX	Q07812	Q07812	BCL2	P10415	Q07812
BRAF	P15056	P15056	BRCA1	P38398	Q8WX92
EGFR	P00533-4, P00533	P07900, P29353, P22681, P19174, P29350, Q06124, Q03135, P12931, Q05397, P15498, Q05209, P46109, Q99962, Q99963, P08581, P17252, Q07889, Q9UQB8, Q96JA1, O14544, Q13480, P27986, P46108, P40763, P01133, Q13322, Q68CZ2, P31749, Q13905, P30530, Q9UQC2, Q9UNE7, P46934, P16234, Q99952, P41240, Q16620, P00533, P04792	IL4	P05112	P31785
JAK1	P23458	O60674, P40763	JAK2	O60674	O60674
JAK3	P52333	Q9UNE7	KRAS	P01116	P15056, P61586
MUC1	P15941	P12931, P00533	Myc	EBI-1265559, P01106	Q14839, P04792, P40763
PIK3CA	P42336	P27986, P35568, P42336	STAT	EBI-10952519	P40763
STAT3	EBI-9914958, P40763-2, P40763	P22681, Q9NWQ8, Q9NP31, P49137, P12931, P00533, P06401, P40763	p53	P04637	Q96ST3, Q09472, P04271, P04792, Q05397

Interaction by TGF-Beta family

3 submitted entities found in this pathway, mapping to 4 Reactome entities

Input	UniProt Id	Input	UniProt Id	Input	UniProt Id
MAPK	P28482	Myc	P01106	PARP1	P09874

Input	Ensembl Id
Myc	ENSG00000136997

Interactors found in the analysis (9)

Input	UniProt Id	Interacts with	Input	UniProt Id	Interacts with
BRCA2	P51587	P09874	EGFR	P00533	Q9UNE7, P35813, P22681
IL2	IL2	Q13547	JAK3	P52333	Q9UNE7
Myc	P01106	P37173, Q13547, P08047	NFKB1	P19838-PRO_0000030311, P19838	Q13547, O00255
PARP1	P09874	P09874	STAT3	P40763	P22681, P08047
p53	P04637	Q09472, Q13547, O14980, P08047, Q15796, P09874			

Signaling by NOTCH

3 submitted entities found in this pathway, mapping to 4 Reactome entities

Input	UniProt Id	Input	UniProt Id	Input	UniProt Id
EGFR	P00533	Myc	P01106	p53	P04637

Input	Ensembl Id
Myc	ENSG00000136997

Interactors found in the analysis (4)

Input	UniProt Id	Interacts with	Input	UniProt Id	Interacts with
EGFR	P00533	P31749	IL8	EBI-1566585	Q92793
Myc	P01106	Q8N6T7	p53	P04637	Q09472, Q92793, P49757, Q06330

Wnt signaling

4 submitted entities found in this pathway, mapping to 6 Reactome entities

Input	UniProt Id	Input	UniProt Id
KRAS	P01116-2	Myc	P01106
WNT1	P04628	WNT6	Q9Y6F9

Input	Ensembl Id
Myc	ENSG00000136997, ENST00000377970

Interactors found in the analysis (8)

Input	UniProt Id	Interacts with	Input	UniProt Id	Interacts with
BRAF	P15056	P63104	EGFR	P00533	P17252, P32121, P63104
IL2	IL2	Q13547	Myc	P01106	Q9Y4A5, Q13547, O15169, P49841
NFKB1	P19838-PRO_0000030311, P19838	Q13547, O00255, Q9Y297, P35222	PARP1	P09874	Q9NTX7
STAT3	P40763	Q9UBE8, O43318	p53	P04637	Q9Y265, Q13547, O14980, Q15291, O14641, Q92993, O15169, P49841, P63104, Q9UBL3
