# Supplementary material for: Aspirin and Cancer Survival: An Analysis of Molecular Mechanisms
Source: Cancers (Basel). 2024 Jan 3;16(1):223. doi: 10.3390/cancers16010223 (PMC10778469; doi:10.3390/cancers16010223)
Supplement: Supplementary file 1 [file cancers-16-00223-s001.zip › additional File S5.pdf]

Interactions with apoptotic pathway

Interactors found in the analysis (9)					
BAX	Q07812, Q07813-1	Q07817, Q16611, O43521, P10415, Q07812, P55957	BCL2	P10415, P10415-1	P51572, Q92934, Q16611, O43521, P10415, Q07812, P55957, Q13794, Q9BXH1
EGFR	P00533	P10636, P12830, Q8WUM4, P45983, P06396, Q05397	Myc	P01106	O60313, P06396
NFKB 1	P19838	P35222	PARP1	P09874	P09429
STAT3	P40763	P51813	TNF	P01375	Q13546, Q13490
p53	P04637	P09429, Q05655, P10415, Q8N726, Q05397			
