# Supplementary material for: Aspirin and Cancer Survival: An Analysis of Molecular Mechanisms
Source: Cancers (Basel). 2024 Jan 3;16(1):223. doi: 10.3390/cancers16010223 (PMC10778469; doi:10.3390/cancers16010223)
Supplement: Supplementary file 1 [file cancers-16-00223-s001.zip › Supplementary Files S6¿CS8.pdf]

Supplementary table S6

Interactions with extracelluar matrix pathway

Input	UniProt Id	Interacts with
EGFR	P00533	P12830

Supplementary table S7

Interactions with DNA repair pathway

Interactors found in the analysis (7)					
BRCA 1	P38398, P38398-1	Q9BX63, O15360, Q12888, P51587, P38398, P46736, P40692, Q92560, P52292, Q99728, Q9BXW9, Q96RL1, Q6UWZ7, Q06609, Q86YC2, O14757	BRCA2	P51587	P38398, O43542, P60896, O43502, Q9Y253, Q9BXW9, P51587, Q06609, Q86YC2
EGFR	P00533	P00519, P53041, P07992, P45983	MUC1	P15941	P00519
Myc	P01106	Q8N6T7, Q12888, P52292, P52701	PARP1	P09874	Q8N2W9, P78527
p53	P04637	O96017, P38398, Q09472, P63279, Q12888, Q92993, Q8N2W9, P51587, Q96PM5			

Supplementary table S8

Autophagy pathway

Interactors found in the analysis (4)

Input	UniProtId	Interacts with	Input	UniProtId	Interacts with
BAX	Q07813	P21796	BCL2	P10415	Q9C0C7
BRAF	P15056	P11142	EGFR	P00533	P11142
