# Supplementary figures and images for: Aspirin and Cancer Survival: An Analysis of Molecular Mechanisms
Source: Cancers (Basel). 2024 Jan 3;16(1):223. doi: 10.3390/cancers16010223 (PMC10778469; doi:10.3390/cancers16010223)

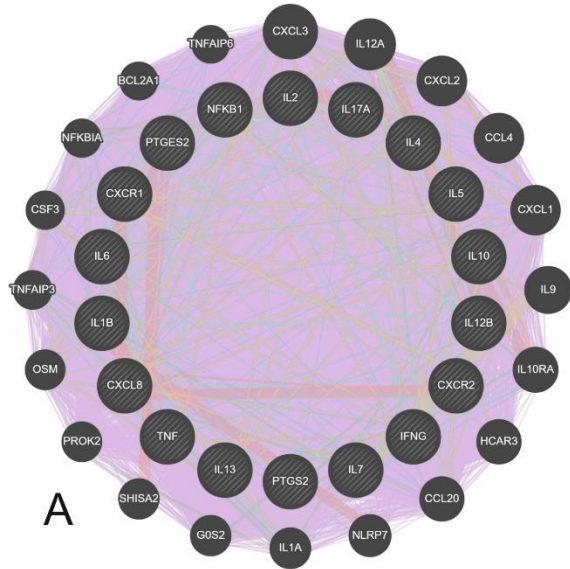

Supplement: Supplementary file 1 [file cancers-16-00223-s001.zip › Additional Figure S1.pdf]
