# Supplementary material for: Aspirin and Cancer Survival: An Analysis of Molecular Mechanisms
Source: Cancers (Basel). 2024 Jan 3;16(1):223. doi: 10.3390/cancers16010223 (PMC10778469; doi:10.3390/cancers16010223)

Additional Figure S2: A). The gene-gene interaction of PTGS2 and PTGES2 with tumor suppressor genes (purple – co-expression, pink -physical interaction, green-genetic interaction, orange-predicted interactions). B). protein-protein interaction of PTGS2/PTGES2 with tumor suppressor proteins having 5 nodes and 5 edges, the interaction was statistically not significant (p=0.07). (light blue- curated databases, pink-experimentally determined; green- predicted gene neighborhood; red-predicted gene fusion; blue-predicted gene co-occurrence). C) Protein-protein interaction of Cellular proliferation and tumor oncogenes with tumor suppressor genes showing statistically significant interactions ($p=<1.0e^{-16}$)

(light blue- curated databases, pink-experimentally determined; green- predicted gene neighborhood; red-predicted gene fusion; blue-predicted gene co-occurrence)

. D) Protein-protein interaction of interleukin and cytokine genes with tumor suppressor genes showing statistically significant interactions ($p=<1.0e^{-16}$)

(light blue- curated databases, pink-experimentally determined; green- predicted gene neighborhood; red-predicted gene fusion; blue-predicted gene co-occurrence)

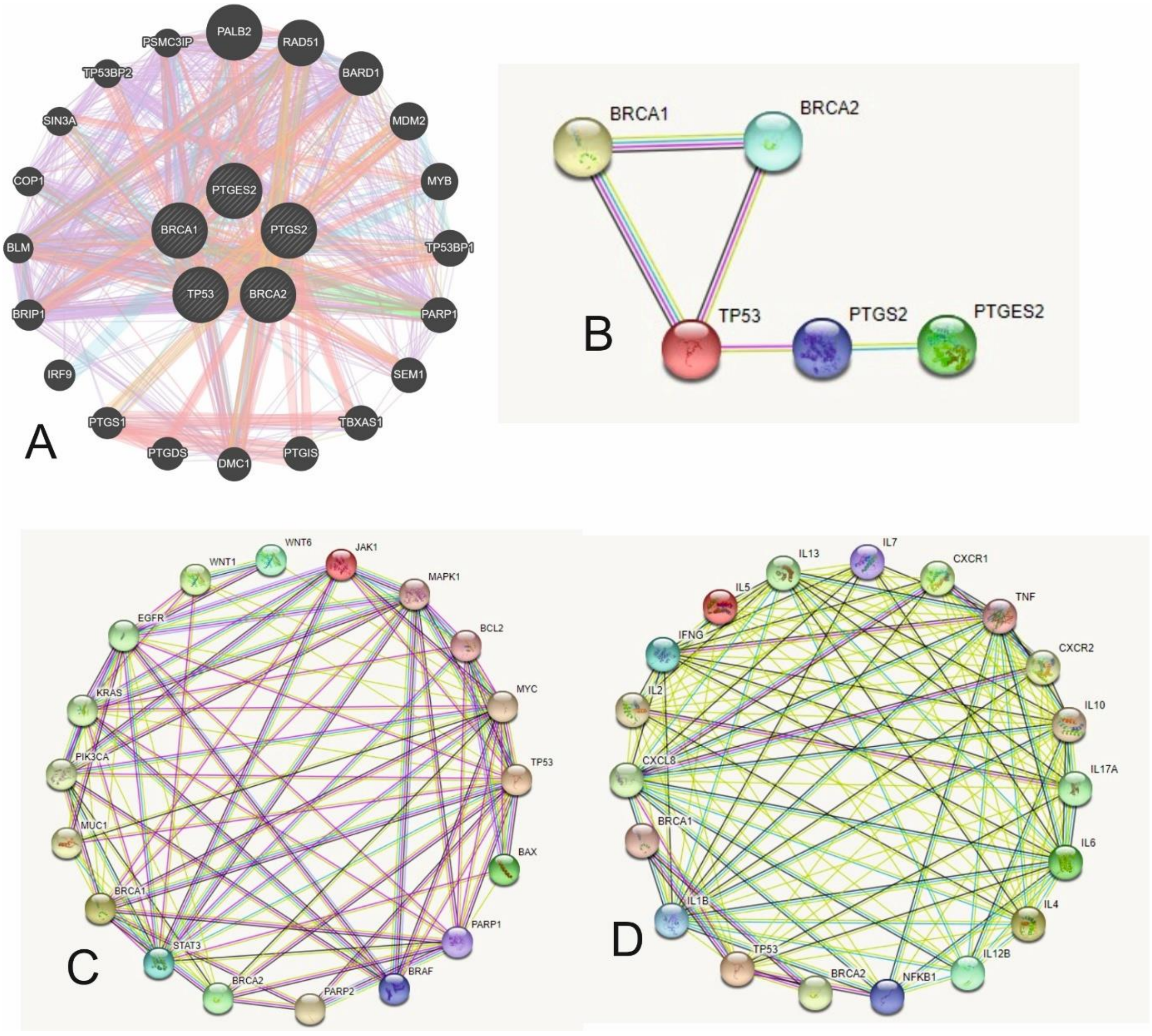

Supplement: Supplementary file 1 [file Additional Figure S2.pdf › Additional Figure S2.pdf]
